# Supplementary material for: Plant-Produced S1 Subunit Protein of SARS-CoV-2 Elicits Immunogenic Responses in Mice
Source: Vaccines (Basel). 2022 Nov 18;10(11):1961. doi: 10.3390/vaccines10111961 (PMC9695278; doi:10.3390/vaccines10111961)
Supplement: Supplementary file 1 [file vaccines-10-01961-s001.zip › vaccines-1961976-supplementary.pdf]

Supplementary Information

Plant-Produced S1 Subunit Protein of SARS-CoV-2 Elicits Immunogenic Responses in Mice

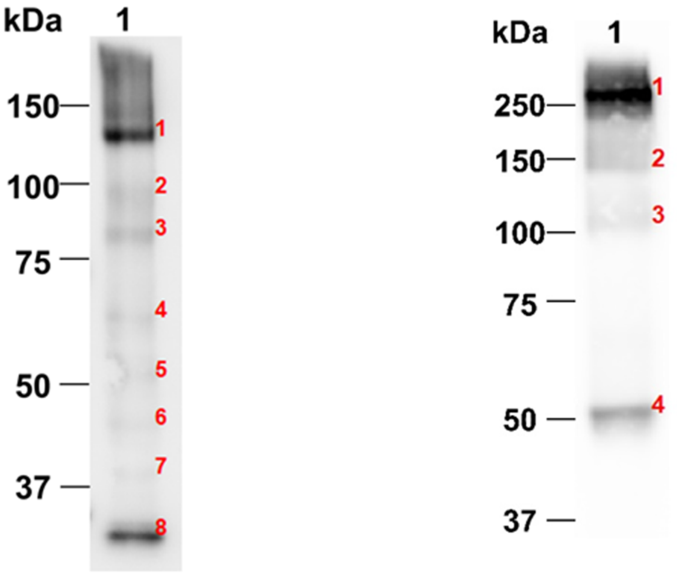

(a) **(b)**

Reducing Condition			
**Band**	**Protein Loaded (μg/Well)**	**Intensity**	**Ratio**
1	5	8704176	0.3316
2	5	2396628	0.0913
3	5	2138682	0.0815
4	5	1648608	0.0628
5	5	1679418	0.0640
6	5	1449006	0.0552
7	5	1351662	0.0515
8	5	6882798	0.2622
**Non-Reducing Condition**			
**Band**	**Protein Loaded (μg/Well)**	**Intensity**	**Ratio**
1	1	35865648	0.6220
2	1	7453082	0.1292
3	1	4411407	0.0765
4	1	9935289	0.1723

Figure S1. The western blot and densitometry readings/intensity ratio of plant-produced SARS-CoV-2 S1-Fc protein. The SARS-CoV-2 S1-Fc protein was purified by single-step protein A affinity chromatography and the purified plant-produced SARS-CoV-2 S1-Fc was analyzed by western blot analysis under reducing (**a**) and non-reducing conditions (**b**), respectively.

Table S1. SARS-CoV-2 RBD-specific T-cell responses in immunized mice. Mouse splenocytes were stimulated with RBD peptide pools and analyzed by mouse IFN-γ ELISpot assay. Data presented as mean ± SD (n = 5). SFCs: Spot-forming cells.

Group	Vaccination	Sample No.	IFN-γ ELISpot (SFU/10^6 Splenocyte)					
			Negative	Positive	SARS-CoV-2-Pool#3	SARS-CoV-2-Pool#4	SARS-CoV-2-Pool#5	Total
1	PBS + Alum	1	0	2496	0	0	0	0
		2	0	2446	0	0	0	0
		3	0	578	0	0	0	0
		4	0	2726	0	0	0	0
		5	0	2616	0	0	0	0
							Mean ± SD	0 ± 0
2	10 μg SARS-CoV-2 S1-Fc + Alum	1	0	2244	0	0	172	172
		2	0	1952	22	0	0	22
		3	0	0	0	0	2	2
		4	0	2432	90	32	112	234
		5	0	2268	40	138	54	232
							Mean ± SD	132.4 ± 112.9
